# Astragalin: A Bioactive Phytochemical with Potential Therapeutic Activities

**DOI:** 10.1155/2018/9794625

**Published:** 2018-05-02

**Authors:** Ammara Riaz, Azhar Rasul, Ghulam Hussain, Muhammad Kashif Zahoor, Farhat Jabeen, Zinayyera Subhani, Tahira Younis, Muhammad Ali, Iqra Sarfraz, Zeliha Selamoglu

**Affiliations:** ^1^Department of Zoology, Faculty of Life Sciences, Government College University, Faisalabad 38000, Pakistan; ^2^Department of Physiology, Faculty of Life Sciences, Government College University, Faisalabad 38000, Pakistan; ^3^Department of Biochemistry, University of Agriculture, Faisalabad 38000, Pakistan; ^4^Department of Medical Biology, Faculty of Medicine, Nigde Ömer Halisdemir University, Nigde 51240, Turkey

## Abstract

Natural products, an infinite treasure of bioactive chemical entities, persist as an inexhaustible resource for discovery of drugs. This review article intends to emphasize on one of the naturally occurring flavonoids, astragalin (kaempferol 3-glucoside), which is a bioactive constituent of various traditional medicinal plants such as *Cuscuta chinensis*. This multifaceted compound is well known for its diversified pharmacological applications such as anti-inflammatory, antioxidant, neuroprotective, cardioprotective, antiobesity, antiosteoporotic, anticancer, antiulcer, and antidiabetic properties. It carries out the aforementioned activities by the regulation and modulation of various molecular targets such as transcription factors (NF-*κ*B, TNF-*α*, and TGF-*β*1), enzymes (iNOS, COX-2, PGE2, MMP-1, MMP-3, MIP-1*α*, COX-2, PGE-2, HK2, AChe, SOD, DRP-1, DDH, PLC*γ*1, and GPX), kinases (JNK, MAPK, Akt, ERK, SAPK, I*κ*B*α*, PI3K, and PKC*β*2), cell adhesion proteins (E-cadherin, vimentin PAR-2, and NCam), apoptotic and antiapoptotic proteins (Beclin-1, Bcl-2, Bax, Bcl-x_L_, cytochrome c, LC3A/B, caspase-3, caspase-9, procaspase-3, procaspase-8, and IgE), and inflammatory cytokines (SOCS-3, SOCS-5, IL-1*β*, IL-4, IL-6, IL-8, IL-13, MCP-1, CXCL-1, CXCL-2, and IFN-*γ*). Although researchers have reported multiple pharmacological applications of astragalin in various diseased conditions, further experimental investigations are still mandatory to fully understand its mechanism of action. It is contemplated that astragalin could be subjected to structural optimization to ameliorate its chemical accessibility, to optimize its absorption profiles, and to synthesize its more effective analogues which will ultimately lead towards potent drug candidates.

## 1. Introduction

Medicinal plants have been an infinite source of therapeutic agents since millions of years. Most of the discovered drugs either belong to natural products or derivatives of natural compounds [1, 2]. The actual fact is that nature is the creator of seemingly limitless series of molecular structures. These structures can serve as unlimited sources for the development of drugs, robust chemotypes, and pharmacophores which are able to be amplified into scaffolds of novel drugs for the cure of various ailments [3]. Before the advent of the postgenomic era with high throughput screening, approximately 80% of drugs were either pure extracts of medicinal plants or the semisynthetic analogues of various compounds from natural sources [4]. After the second world war, the pharmaceutical research expanded to massive screening of plant extracts in search of new drugs from natural resources [5]. To date, about 61% of anticancer and 49% of anti-infective compounds have been discovered from natural products [6].

The term “natural products” encompasses chemical entities derived from plants, bread molds, microorganisms, terrestrial vertebrates as well as invertebrates, and marine organisms [7]. These chemical entities are known to have immense chemical diversity with outstanding drug-like properties that contribute towards their multitargeted action [8]. A lot of plant-derived bioactive compounds are used for the cure as well as for the prevention of several diseases. Among these compounds are the polyphenols consisting of alcohols with ≥2 benzene rings and ≥1 hydroxyl group. These polyphenols have a range from simple structural molecules (flavonoids and phenylpropanoids) to highly complex compounds (lignins and melanins). Reports have suggested that polyphenols in general and flavonoids in particular exhibit various biological effects like antiallergic, antibacterial, anti-inflammatory, antiviral, antithrombic, hepatoprotective, antibacterial, and antioxidant activities [9].

Flavonoids are structurally diverse and most abundantly found polyphenols in the human diet [10]. They are mostly found in the form of glycosides and acylglycosides. Flavonoids have been divided into various classes such as flavones, flavonols, flavanones, flavanonols, flavanols or catechins, and anthocyanins. They are the essential constituents of our food and are found in onions, parsley, berries including blue berries, black tea, green tea, bananas, red wine, all citrus fruits, sea blackthorns, and dark chocolates with the contents of 70% or more [11].

Astragalin (kaempferol-3-*O*-*β*-D-glucoside), a bioactive natural flavonoid, has been well known for its medicinal importance. It has been reported to exhibit multiple pharmacological properties including antioxidant [12, 13], anti-inflammatory [14], anticancer [15], neuroprotective [16], and cardioprotective property [16].

## 2. Natural Sources of Astragalin

**Figure 1:**
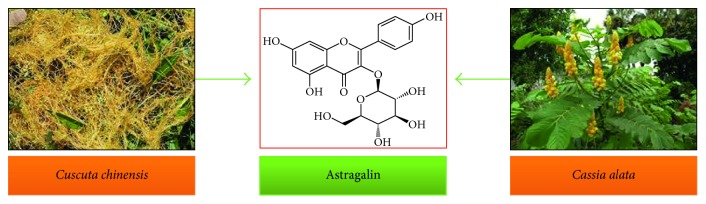
Natural sources of astragalin.

Astragalin, a naturally occurring flavonoid, has been identified in a variety of plants (Figure 1 and Table 1) such as *Cuscuta chinensis* Lam., a member of the *Convolvulaceae* family, which consists of about 60 genera and 1,650 species. The seeds of the genus *Cuscuta* are a rich source of astragalin and are utilized as a traditional folk medicine to cure osteoporosis in various Asian countries including Pakistan [17]. *C. chinensis* has high contents of astragalin, that is, 29–34% of total phenolics as compared to other species [18]. *Cassia alata* belongs to the family *Fabaceae* (the largest family among angiosperms) that comprises of ∼700 genera and 20,000 species. The leaves of *C. alata* are found to be effective against skin diseases including eczema and chronic skin impurities in tropical regions of the world (Malaysia, Brazil, and Indonesia) [19]. Astragalin has also been isolated from the plants of *Ebenaceae, Rosaceae*, and *Eucommiaceae* families. The summary of plants containing astragalin, parts utilized, and biological features are enlisted in Table 1.

Astragalin can also be produced *in vivo* by glycosylation of kaempferol at the 3C-O position [20]. UDP-dependent glycosyltransferases (UGT) were used as biocatalysts in the synthesis of astragalin. A recombinant strain of *Arabidopsis thaliana* was used to construct an efficient UDP-glucose synthesis pathway by use of enzymes such as uridylyltransferase, sucrose phosphorylase, and sucrose permease. BL21-II was a recombinant strain designed to scale up the production of astragalin by using a fed-batch fermentator.

## 3. Biological Activities of Astragalin and Their Mechanisms of Action

**Figure 2:**
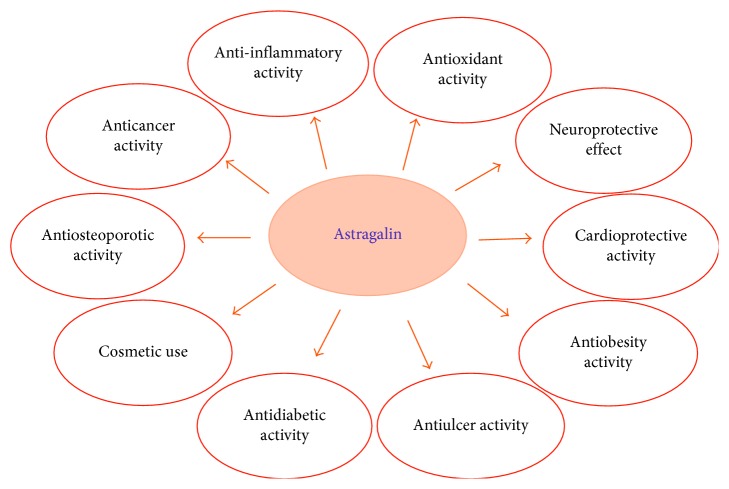
Biological activities of astragalin.

The biologically active and therapeutically effective compound “astragalin” has been known to possess broad spectrum of pharmacological features such as anticancer, anti-inflammatory, antioxidant, neuroprotective, antidiabetic, cardioprotective, antiulcer, and antifibrotic as shown in Figure 2. Various *in vivo* and *in vitro* investigations on astragalin have elucidated its medicinal characteristics and mechanism of actions.

### 3.1. Anti-inflammatory Activity

Inflammation is an immediate response of a body to tissue damage caused by pathogens and toxic stimuli such as physical or chemical injury. Although inflammatory response is a defense mechanism, but if persistent, it can lead to multiple pathological conditions such as cancer, allergy, atherosclerosis, and autoimmune diseases [119]. Negative after effects associated with nonsteroidal type anti-inflammatory drugs (NSAIDs) arouse a need among researchers to find out effective and safe alternatives [120]. Plant extracts enriched with flavonoids have been known to possess anti-inflammatory activity [121].

Astragalin, a bioactive natural flavonoid, has been known to mitigate inflammation in LPS-induced murine model of mastitis and lung injury model via reducing the activity of myeloperoxidase and the expression of IL-1*β*, IL-6, and TNF-*α*. Astragalin's anti-inflammatory response proceeds via inhibition of LPS-induced activation of NF-*κ*B, as it is actively involved in alleviating the deterioration of IkB*α* and restricting the nuclear translocation of NF-*κ*B [92, 122]. Another investigation on LPS-stimulated expression of inflammatory mediators in macrophages has declared the fact that astragalin actively inhibited the expression of proinflammatory mediators via inhibiting NF-*κ*B signaling pathway [123]. Astragalin has been known to halt the MAPK and NF-*κ*B pathways in leptospira-induced uterine and epithelial inflammation in mice [124]. Astragalin has capability to inhibit the production of prostaglandin E2 (PGE2) in periodontal pathogen-induced periodontitis, a destructive inflammatory pathological condition, in human gingival epithelial cells [125]. Astragalin has been investigated to determine the underlying mechanism for its protective effect against ovalbumin-stimulated allergic reactions in mouse models of allergic asthma. Results have declared that it effectively lowers the eosinophil count in lung tissues and inhibited eosinophilia induced by ovalbumin. As a result, IgE, IL-4, IL-5, and IL-13 were retrieved in bronchoalveolar lavage fluid [126]. Purely prepared astragalin inhibited the activity of PGE2 and downregulated the production of cellular nitrite oxide and IL-6 in LPS-stimulated RAW 264.7 cells [33]. Astragalin treatment leads to the inhibition of alveolar destruction, allergic inflammation, and thickening of airways in the ovalbumin-induced inflammatory mouse model [14]. Anti-inflammatory activities of astragalin in different animal models are recorded in Table 2.

### 3.2. Antioxidant Activity

In living systems, free radicals such as hydroxyl radicals (OH·), superoxide anion (O_2_·−), singlet oxygen (^1^O_2_), and ROS are reported to have deleterious impacts on cellular functions. Excessive production of free radicals may affect the balance of prooxidant and antioxidant systems in the body, thus causing various pathological conditions such as arterial hypertension, rheumatism, inflammation, diabetes, cancer, neurodegenerative disorders, and genetic mutations [120]. Researchers have affirmed various plant extracts as natural and infinite treasure of antioxidants. These antioxidants act as free radical scavengers, electron donors, and chelating agents for free catalytic metals in biological systems [75].

Astragalin also inhibits the endotoxin-induced oxidative stress, which can lead to epithelial apoptosis and eosinophilia. It can also act as an antagonizing agent against endotoxin-induced oxidative stress via modulation of LPS-TLR signaling network [129]. Astragalin causes the suppression of 6-hydroxydopamine-stimulated neurotoxicity in *Caenorhabditis elegans* via modulation of apoptosis-related pathways and alleviation of oxidative stress [130]. Astragalin has capability to improve neural function in the ischemia brain injury model of rats via blocking the apoptosis in the hippocampus region by enhancing the expression of NCam [131] (Table 3).

### 3.3. Neuroprotective Activity

Disturbance in cerebral redox homeostasis is the main cause of neurodegenerative diseases in humans. Cerebral oxidative stress leads to dopaminergic neuronal cell death and dysfunction. Neuroprotective mechanism of naturally occurring bioactive entities is associated with their free radical scavenging capability generated by neurotoxins and oxidative stress-induced processes in neuronal cells of the brain [133].

Astragalin has been reported to decrease the neurodegeneration in *C. elegans* stimulated by 6-OHDA and increase lifespan of astragalin-treated nematode. It also reduces the ROS levels, inhibits lipid peroxidation, and increases SOD and GPx activities. Furthermore, it is capable of enhancing AChE and reducing the transcript level of proapoptotic gene *egl-1* associated with neuronal cell death [130]. In another attempt, the effects of astragalin on CNS were assessed by the application of the leaves extract of *Eucommia ulmoides.* The extract with high percentage of astragalin had a significant effect on metabolism of mice. Moreover, it effectively prolonged the convulsion latency and diminished the convulsion rate. These results strengthen the fact that *E. ulmoides* has a very good hypnotic effect on CNS [49]. Astragalin also suppressed carrageenan-stimulated paw edema in rats. Neural function is also reported to be improved by the use of astragalin in ischemic brain injury rat models [131].

### 3.4. Cardioprotective Activity

Myocardial infarction and ischemic heart failure are the leading causes of mortality in the developing countries, and their number is increasing day-by-day. They may result in reperfusion arrhythmias, myocardial stunning, and similar other cardiovascular disorders [16]. An enhanced perception of ischemia reperfusion (I/R) damage provides an innovative approach for new cardioprotective administrations [134]. Regulation of bradykinin, adenosine, opioid, adrenergic, and other G-protein connected receptors have been known to be associated with myocardial protection [135].

Certain epidemiological studies have confirmed that flavonoids stimulate cardioprotective effects against myocardial ischemia [136]. Astragalin, a bioactive flavonoid, was proved to be effective against acute I/R injury in Sprague-Dawley rats as its mechanism of action precedes via diminishing intracellular oxidative stress and apoptosis. The associated mechanism involves decreased expression of MDA, TNF-*α*, IL-6, ROS, and Bax along with the increased ratio of GSH/GSSG, respectively [137].

### 3.5. Antiobesity Activity

The term “obesity” can be defined as impaired energy balance that usually results from either enhanced caloric intake and/or reduced energy consumption. Currently, much attention has been given to several nutritional aspects that may be useful for inhibiting body fat accumulation and decreasing the risk of diseases related to obesity. In case of mammals, energy metabolism is maintained by lipolytic action in adipose tissues which is generally stimulated by some pharmacologically important lipolytic hormones such as nor-epinephrine, epinephrine, and catecholamines [80]. Many cellular investigations have determined that dietary polyphenols decrease viability of adipocytes and growth of preadipocytes, downregulate triglyceride accumulation and adipocyte differentiation, and induce fatty acid beta-oxidation and lipolysis [138].

Astragalin along with other known flavonoids isolated from *N. nucifera* showed inhibitory effect on diet-induced obesity and also activated *β*-adrenergic receptor pathway, but additional experimentation is required to fully elucidate its possible mechanism of action [80].

### 3.6. Antiulcer Activity

Ulcer is a chronic lesion which usually develops due to an imbalance between numerous protective and aggressive factors. Gastric ulcers being represented by repeated incidents of healing and reexacerbation contribute towards chronic inflammation which may persist for 10–20 years. It is a well-known fact that naturally occurring phenolic entities have capability to shield gastric mucosa from injury due to their cytoprotective and antioxidant features. Furthermore, flavonoids stimulate mucus secretion, block pepsinogen, prohibit Ca^2+^ influx, and also change GSH metabolism. Astragalin, a pharmacologically active flavonoid isolated from *C. cyparissias*, has been examined for its antiulcer activity. Results demonstrated that 30 mg/kg dosage of astragalin effectively decreases percentage of lesion area, total area of lesion, and ulcer index in the mice model of gastric secretion [97].

### 3.7. Antidiabetic Activity

Diabetes mellitus is characterized by hyperglycemia which is caused by deficit in insulin action or production [139]. Currently available antidiabetic therapeutics such as hypoglycemic drugs and insulin have limitations of their own. Natural products and herbal medicines have been suggested as one of the treatment options for diabetes since ancient times. Naturally occurring bioactive chemical entities such as flavonoids, terpenoids, alkaloids, and phenolics have been reported as antidiabetic agents [140].

Diabetic retinopathy (DR) arises due to diabetes mellitus and is one of the most common causes of vision loss. Hyperglycemia leads to overexpression of many biological effectors such as vascular endothelial growth factor (VEGF) which is very crucial for the development of DR. Astragalin derived from *A. membranaceus* has beneficial effects against hyperglycemia. It helps to prevent DR by decreasing the overexpression of VEGF in cultured muller cells and alleviating the effects caused by high concentration of glucose in the blood [141].

### 3.8. Antifibrotic Activity

Environmental factors like air pollutants may result in considerable production of reactive oxygen species in the airways. Astragalin isolated from leaves of persimmon and green tea can be effectual in allaying ROS-prompted bronchial fibrosis as it has capability to inhibit auto phagosome formation in the airways [132]. It also alleviates hepatic fibrosis by regulating PAR2 (protease-activated receptor 2) mechanism. AGS regulates proinflammatory cytokines namely IL-6, IL-1*β*, and TNF-*α*. It also attenuates the PAR2 signaling expression, and its protective effects are especially prominent in diabetic animal models [128].

### 3.9. Cosmetic Use

Astragalin glucosides can be used as valuable agents in cosmetics due to their important chemical characteristics. First of all, it inhibits collagenase activity. Collagenase is involved in the hydrolyzation of dermal matrix protein formation as well as wrinkle formation. Secondly, astragalin has an antioxidant activity as it alleviates the free radical species. Thirdly, astragalin controls the pigmentation in the skin caused by melanin [142]. Melanin pigment causes darkening of complexion in skin, eyes, and hair in humans. *Nelumbo nucifera* (lotus) contains bioactive compounds astragalin and hyperoside in the receptacles which are known to be the melanogenesis inhibitor, thus possibly decreasing the skin darkness [143]. Astragalin along with quercetin is known to possess protective effect against the UV radiations. UV radiations can make the skin of animals prone to various biological responses such as DNA damage, formation of sunburn cells, melanogenesis, photoaging, skin cancer, hyperplasia, immune suppression, and edema. UV radiations from the sun can also damage macromolecules in the epidermal layer of animals creating specific changes in the skin, for example, mutations in genes and changes in the immune system. Expression of major CXC chemokines, that is, chemokine ligand 1 (CXCL1) and chemokine ligand 2 (CXCL2), at sites of inflammation within the skin are upregulated after the exposure of skin to UV radiations. These chemokines are the potent stimulators of neutrophil activation which later on produce ROS and leads to oxidative stress. Astragalin, a major flavonoid, can be used as a barrier against UV-induced damage as it is associated with downregulation of CXCL-1 and CXCL-2 in the skin and thus can be used as a photoprotective agent [144] (Table 4).

### 3.10. Antiosteoporotic Activity

Osteoporosis is characterized by structural deterioration of tissues in the bone along with lower bone mass and bone fragility. The main causes of osteoporosis include estrogen deficiency, excess of glucocorticoids, and oxidative stress. Astragalin, an active compound, isolated from crude methanolic extract of the seeds of *C. chinensis* showed estrogenic activity against osteoporosis, and it is responsible for significant osteoblastic cell proliferation in UMR-106 osteoblastic cells [17].

### 3.11. Anticancer Activity

Currently, cancer is the second leading cause of mortality worldwide. In spite of advances in the development of new therapeutic preferences for cancer, its ratio is increasing day by day. Every year, almost 7 million people die due to cancer. Lung cancer particularly non-small cell lung cancer (NSCLC) accounts for more than 80% of deaths all around the world today. Therefore, it is necessary to discover new cheap and inexpensive drugs that can ameliorate the antitumor effects and reduce the side effects of generally recommended chemotherapy drugs [145].

Natural phytochemicals that are active constituents of medicinal plants, seeds, fruits, and herbs including polyphenols (flavonoids, terpenoids, and carotenoids) have gained significant recognizance for their potential value as therapeutic agents [146, 147]. Much research work has been conducted towards the assessment of phenolic phytochemicals as potent prophylactic agents as they can act on multiple cellular targets. The mechanistic insight into chemoprevention incorporates induction of apoptosis and cell cycle arrest or prohibition of certain cell signaling pathways mostly protein kinases C (PKC), glycogen synthase kinase (GSK), mitogen-activated protein kinases (MAPK), and phosphoinositide 3-kinase (PI3K) leading to abnormal AP-1, COX-2, and NF-*κ*B expressions. Efficacy of chemopreventive agents revert their capacity to counteract with certain up-stream signals that leads to redox imbalances, genotoxic injury, and other situations of cellular stress. Thus, targeting damaged molecules along with interrupted signal transduction pathways in cancer epitomize a rational strategy for chemoprevention, and phenolic compounds seem to be auspicious in this aspect [147, 148]. In recent years, flavonoids have drawn developing consideration as powerful anticancer agents against various cancer types [149].

Several investigations on astragalin have explained its anticancer effect due to its promising competency to inhibit proliferation in different cancer cell lines including leukemia (HL-60) [15], hepatocellular (HepG2, Huh-7, and H22) [150], skin (HaCaT, A375P, and SK-MEL-2) [151], and lung (A549 and H1299) cancerous cells [145].

Astragalin heptaacetate (AHA), a therapeutically active flavonoid, induces apoptosis in HL-60 cells through release of cytochrome c into the cytosol. The associated mechanism involves activation of Bax, caspase-3/-7, and p38MAPK and intracellular ROS generation along with inhibition of cell signaling pathways JNK/SAPK and ERK ½ [15]. Astragalin also prohibits TNF-*α*-induced NF-*κ*B activation in A549 and H1299 cells. Moreover, AG-triggered cell death is affiliated with increased Bax : Bcl-2 ratio and enhanced cleavage of caspase-3/-9 and PARP in conjunction with blockage of PI3K/Akt, MAPK, and ERK 1/2 signaling cascades in a time- and dose-related manner [145]. In hepatocellular carcinoma cells, astragalin (AG) significantly suppressed proliferation both *in vitro* in HepG2 cells and *in vivo* in Huh-7 (nude mice) and H22 (Kunming mice) cells via mechanistically inhibiting hexokinase 2 and upregulating miR-125b expression, respectively [150].

Astragalin can be a novel anticancer agent for the cure and prevention of UVB-stimulated actinic keratosis skin lesion by suppressing phospho-MSK1, *γ*-H2AX, and p38MAPK activation in a time-and dose-related manner in human HaCaT cells in vitro and Babl/c mice *in vivo*. In another report, astragalin strongly exerted cytotoxic effects in A375P and SK-MEL-2 cancerous cells in a concentration-dependent way through induction of apoptosis. The underlying cell death mechanism involves activation of Bax and caspase-3/-9, cleavage of PARP, and downregulation of cyclin D1 and Mcl-1 along with inhibition of Sry-related HMg-Box Gene 10 (SOX10) signaling cascade [151, 152]. The reported data recommend astragalin's multitargeted activity in preference to single effect that may perform an imperative role towards developing astragalin into potential anticancer drug in future (Table 5).

## 4. ADMET Profiles of Astragalin

ADMET profiles along with biological activity spectra were performed for astragalin based on in-silico tools. The results indicate that astragalin is a potential anticancer agent which is unlikely to present any acute hazard or toxicity. Furthermore, astragalin can be absorbed by human intestines, but it is incapable of penetration to Caco-2 cells. Astragalin has been validated as a novel substrate of p-glycoprotein which is crucial for the metabolism and clearance of the compounds and for the efflux of drugs [154].

## 5. Conclusions and Future Perspectives

**Figure 3:**
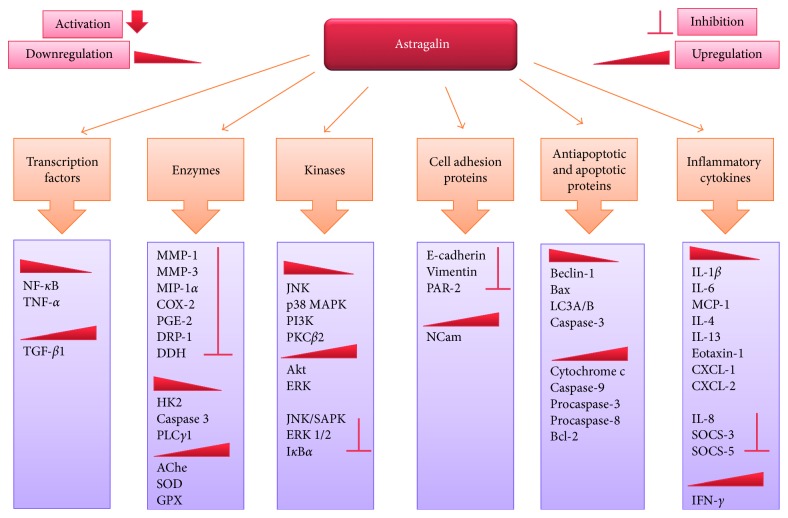
A diagrammatic representation of molecular targets and mechanism of action of astragalin. Astragalin has capability to modulate various transcriptional factors, enzymes, protein kinases, cell adhesion molecules, apoptotic and antiapoptotic proteins, and inflammatory cytokines resulting in anticancer, anti-inflammatory, antioxidant, and cardioprotective activities.

Astragalin, a natural flavonoid, has been isolated from various traditional medicinal plants such as *Cassia alata, Moringa oleifera, Nelumbo nucifera, Cuscuta* spp., *Radix astragali*, *Morus alba*, and *Eucommia ulmoides*. Astragalin has been reported to modulate inflammatory responses by regulating the expression of NF-*κ*B, iNOS, cytokines/chemokines (COX-2, TNF-*α*, IL-10, and IL-6), MAPK signaling pathways (PGE2, IgE, IL-4, IL-5, IL-13, IL-1*β*, and IL-6), and PAR2 signaling expression. It also has the capability to alleviate the production of ROS and inhibit the endotoxin-induced oxidative stress (Figure 3). Astragalin is also known to be an inhibitor of ERK-1/2 and Akt signaling; therefore, it is a significant compound against cancer proliferation. In this review paper, we have emphasized on various pharmacological properties of astragalin such as anti-inflammatory, antioxidant, neurological, cardioprotective, antidiabetic, and anticancer. Although several *in vitro* and *in vivo* investigations have demonstrated its diversified pharmacological applications, further experimentation along with medicinal chemistry approaches and preclinical trials is still obligatory to uncover the knowledge of its biological and pharmacological applications and their associated mechanisms of actions for the treatment and prevention of several diseases.

## Figures and Tables

**Table 1:** Plants containing astragalin as an important constituent with its biological properties.

Name of the plant		Parts used/extract	Biological activities	References
Botanical name	Common name			
*Acer truncatum*	Shantung maple	—	—	[21]
*Aceriphyllum rossii*	Mukdenia	Aerial parts	Antioxidant	[22]
*Agrimonia pilosa*	Hairy agrimony	Aerial parts	Antihemorrhagic, antiplatelet, antioxidant, and acetylcholinesterase inhibitory	[23]
*Allium ursinum*	Wild garlic	Flowers	Antimicrobial	[24]
*Allium victorialis*	Alpine leek	—	Antitumor	[25]
*Alsophila spinulosa*	Hook tryon	Leaves	Antixanthine oxidase	[26]
*Apocynum venetum*	Luobuma	Leaves	Lower blood pressure, antidepressant, antinephritis, and antineurasthenia	[27]
*Jasminum subtriplinerve* Blume	—	Aerial parts	—	[28]
*Astragalus hamosus*	Dwarf yellow milk vetch	Aerial parts	—	[29]
*Caesalpinia decapetala*	Mysore thorn	Leaves	—	[30]
*Calligonum polygonoides*	Phog	Aerial parts	Antiulcer, anti-inflammatory, hypoglycemic, and antioxidant	[31]
*Camellia sinensis*	Tea	Leaves and seeds	Antidysentery, antihyperlipidemia, antihyperglycemia, and anti-inflammatory	[32–35]
*Carthamus lanatus* L.	Downy safflower	Aerial parts	Antioxidant	[35]
*Cassia alata*	Ringworm bush	Leaves	Antioxidant, anti-infectious, and DNA repair	[19]
*Celastrus gemmatus* Loes	Chinese bittersweet	Leaves	—	[36]
*Centella asiatica*	Asiatic pennywort	Leaves	Anti-inflammatory	[37]
*Clerodendrum philipinum*	Chinese glory bower	Roots	—	[38]
*Conyza filaginoides*	Laennecia filaginoides	Aerial parts	Antiprotozoal	[39]
*Corchorus olitorius* L.	Moroheiya	Leaves	Inhibits the histamine	[40]
*Cuscuta chinensis*	Chinese dodder	Seeds	Antiosteoporotic	[17, 41–43]
*Cuscuta australis*	Australian dodder	Seeds	—	[17, 41–43]
*Diodia teres*	Buttonweed	Whole plant	—	[44]
*Drosera peltata*	Sundew		Antitussive	[45]
*Dianthus barbatus* cv	Sweet William	Aerial parts	Anti-inflammatory	[46]
*Eucommia ulmoides*	Hardy rubber tree	Leaves	Antidiabetic, antioxidant, and hypnotic effect	[47–49]
*Eupatorium cannabinum* L.	Hemp agrimony	Aerial parts	—	[50]
*Eupatorium lindleyanum*		Aerial parts	—	[51]
*Exochorda racemosa*	Pearlbrush	—	—	[52]
*Flaveria bidentis* (L.) Kuntze	Coastal plain yellow tops	Leaves	—	[53, 54]
*Flos gossypii*	—	Flowers	—	[55]
*Gladiolus gandavensis*	Gladiolus	Aerial parts	—	[56]
*Glycyrrhiza glabra*	European licorice	Leaves	—	[57]
*Glycyrrhiza uralensis* Fisch	Chinese licorice	Leaves	—	[58]
*Gynura procumbens*	Longevity spinach	—	Antidiabetic	[59]
*Hedera helix*	English ivy	—	—	[60]
*Helianthemum glomeratum*	Island rushrose	Aerial parts	—	[61]
*Hemistepta lyrata* Bunge	—	Whole plant	—	[62]
*Hippophae rhamnoides* L.	Sea buckthorn	Leaves	—	[63]
*Ipomoea batatas*	Sweet potato	Leaf	—	[64]
*Koelreuteria paniculata*	Golden rain tree	Flowers	Antioxidant	[65]
*Allium ampeloprasum*	Wild leek	Leaves	Antioxidant	[66]
*Ligusticum chuanxiong*	—	Aerial parts	—	[67]
*Lindera aggregate*	Evergreen lindera	Leaves	—	[68]
*Litsea coreana*	—	Leaves	Antioxidant	[69]
*Magnolia fargesii*	—	Flowers	Anticomplement	[70]
*Moringa oleifera* Lam.	Drumstick tree	Leaves	Antioxidant	[71]
*Morus alba* L.	White mulberry	Leaves	Hypoglycemic and antioxidant	[72–78]
*Mussaenda arcuate*	Forest star	Leaves		[79]
*Nelumbo nucifera*	Sacred lotus	Leaves	Lipolytic activity	[80–84]
*Ochradenus baccatus*	Taily weed	Aerial parts	—	[85]
*Orostachys japonica*	Rock pine	—	Calpain inhibitory activity	[86]
*Diospyros kaki*	Japanese persimmon	Leaves	Angiotensin converting enzyme activity, and inhibition of atopic dermatitis (AD)	[12, 87–89]
*Rosa agrestis*	Field briar	Leaves	Anti-inflammatory and antioxidant	[13, 90–92]
*Peucedanum alsaticum*	—	Fruits	—	[93]
*Phaseolus vulgaris* L.	Common bean		—	[94]
*Phlomis spinidens*	—	Aerial parts	Antiallergic	[95]
*Phyllanthus muellerianus*	—	Leaves	Antibacterial and anti-inflammatory	[96]
*Polygala cyparissias*	—		Antiulcer	[97]
*Polygonum salicifolium*	Knotweed	Aerial parts	DPPH-free radical scavenging activity	[98]
*Prunus padus* L.	European bird cherry	Flowers and leaves	Antioxidant	[99]
*Prunus serotina* Ehrh	Black cherry	Leaves and flowers	—	[100]
*Pseudotsuga menziesii*	Oregon pine	Needles	Cytotoxic	[101]
*Radix astragali*	Milk vetch root	Roots	Antidiabetic	[102–104]
*Rhus sylvestris*	Sumach	Stems and leaves	Antiosteoporotic	[105]
*Rosa soulieana*	Shrub rose	Flowers	Antioxidant	[106]
*Rubus rigidus* var. *camerunensis*	Ronce blanche	Aerial parts	Antioxidant	[107]
*Sapium sebiferum*	Chinese tallow	Leaves	—	[108]
*Solenostemma argel*	Arghel	Aerial parts	Antibacterial	[109]
*Solidago canadensis* L.	Canada goldenrod	—	Antioxidant	[110]
*Sorbus aria* (L.)	Lutescens	Leaves	—	[111]
*Tadehagi triquetrum*	—	Whole plant	Antimicrobial and anti-inflammatory	[112]
*Tiarella polyphylla*	Foam flower	Whole plant	—	[113]
*Trachelospermum jasminoides*	Confederate jasmine	Leaves	Antifungal	[114]
*Urtica cannabina*	—	Fruits	—	[115]
*Vahlia capensis*	—	—	Antibacterial	[116]
*Vicia calcarata*	Few flowered vetch	Aerial parts	Hepatoprotective	[117]
*Wedelia chinensis*	—	Whole plant	Inhibitor of the complement system	[118]

**Table 2:** Anti-inflammatory activities of astragalin *in vitro* and *in vivo*.

Assay	Organism tested	Dose/concentration	Molecular targets	References
LPS-induced mouse mastitis	Mouse mastitis	10, 25, and 50 mg/kg	TNF-*α*^↓^, IL-1*β*^↓^, IL-6^↓^, p65^┴^, and I*κ*B*α*^┴^	[92]
LPS-induced endotoxemia and lung injury in mice	Mice (lung)	25, 50, and 75 mg/kg	TNF-*α*^┴^, IL-1*β*^┴^, and IL-6^┴^	[122]
LPS-induced macrophages in mice	Mouse cells	1–100 *µ*g/mL	iNOS^↓^, COX-2^↓^,TNF-*α*^↓^, IL-1*β*^↓^, IL-6^↓^, MIP-1*α*^↓^, MCP-1^↓^, NF-*κ*B p65^┴^, I*κ*B*α*^┴^, and NO^┴^	[127]
LPS-induced RAW 264.7 cells.	Mice (RAW 264.7 cells)	1, 10, and 100 *μ*M	NO^↓^ and TNF-*α*^↓^	[37]
Inhibitory activity on the histamine release by KU812 cells	KU812 cells	10 to 30 *μ*mol/L	IL-4^↓^, IL-13^↓^, and (IFN-*γ*) no effect	[12]
LPS-induced inflammation in RAW 264.7 cells	Mice (RAW 264.7 cells)		NO^┴^, IL-6^┴^, and PGE2^┴^	[33]
*P. gingivalis*-induced human gingival epithelial (HGE) cells	Human gingival epithelial cells		COX-2^┴^, IL-6^┴^, IL-8^┴^, MMP-1^┴^, MMP-3^┴^, PGE-2^┴^, and IL-4^┴^	[125]
Anti-inflammatory effects on *Leptospira interrogans*-induced inflammatory response	Uterine and endometrial epithelial cells of mice	100 *μ*g/mL	TNF-*α*^┴^, IL-1*β*^┴^, IL-6^┴^, NF-*κ*B^↓^, p38^┴^, p-p38 MAPK^↓^, ERK^┴^, JNK^┴^, and p-p65^↓^	[124]
Protective effects against ovalbumin- (OVA-) induced allergic inflammation	Mouse model of allergic asthma	0.5 mg/kg and 1 mg/kg	SOCS-3^┴^, SOCS-5^┴^, and IFN-*γ*^↑^	[126]
Alleviation in hepatic fibrosis function	Diabetic rats and nondiabetic rats		PAR2^┴^, IL-1*β*^↓^, IL-6^↓^, TNF-*α*^↓^, and TGF-*β*1^┴^	[128]
Prevention from atopic dermatitis	NC/Nga mice	1.5 mg/kg	IgE^↓^	[87]

^↑^Upregulation; ^↓^downregulation; ^┴^inhibition.

**Table 3:** Antioxidant activity of astragalin *in vitro* and *in vivo*.

Assay	Organism tested	Dose/concentration	Molecular targets	References
Free radical-scavenging activity		1, 3, 10, 30, 100, or 300 *µ*g/mL		[107]
Inhibitory activity against autophagy-associated airway epithelial fibrosis	Mice	1–20 *μ*M	E-cadherin^┴^, vimentin^┴^, Beclin-1^↓^, LC3A/B^↓^, EMT^↓^, and TGF-*β*1^┴^	[132]
Apoptotic and eosinophilia amelioration	BEAS-2B cells	1–20 *μ*M	TLR-4^↓^, Eotaxin-1^↓^, PLC*γ*1^↓^, PKC*β*2^↓^, p-p22^↓^, p-47^↓^, JNK^↓^, p38 MAPK^↓^, Akt^↑^, and ERK^↑^	[129]
Suppression of 6-hydroxydopamine-induced neurotoxicity in *Caenorhabditis elegans*	*C. elegans*	2.0 mg/mL	*egl-1* ^↓^, SOD^↑^, GPX^↑^, AChe^↑^, and p38 MAPK^↓^	[130]
Neuroprotective effect against ischemic brain injury	Wister rats	5 mg/kg and 15 mg/kg	NCam^↑^	[131]

^↑^Upregulation; ^↓^downregulation; ^┴^inhibition.

**Table 4:** Cosmetic uses of astragalin.

Assay	Organism tested	Dose/concentration	Molecular targets	References
Inhibition of melanin secretion	*Leuconostoc mesenteroides*	10 mM	MMP-1^┴^	[142]
Protection against UV damage	Mice (BalB/c) and human keratinocyte cells (HaCaT cells)	2.5 mg/kg and 0.25 *µ*M/ml	CXCL-1^↓^ and CXCL-2^↓^	[144]

^↓^Downregulation; ^┴^inhibition.

**Table 5:** Anticancer activities of astragalin *in vitro* and *in vivo*.

Type of cancer	Cell line	Dose/concentration	Molecular targets	References
Leukemia	HL-60	6 ± 1 *µ*M	Bax^↑^, Bcl-2^↓^, caspase-3/-7^Act^, JNK/SAPK^┴^, and ERK 1/2^┴^	[15]
Hepatocellular	HepG2, Huh-7, and H22	—	HK2^┴^ and miR-125b^↑^	[150]
Skin	HaCaT, A375P, and SK-MEL-2	50 and 100 *μ*M/mL	p38 MAPK^↓^, phospho-MSK1^↓^, *γ*-H2AX^↓^, caspase-9/-3^Act^, Bax^Act^, PARP cleavage, cyclin D1^↓^, Mcl-1^↓^, and SOX10^┴^	[151, 152]
Lung	A549, H1299, H226, H838, H23, H1437, H125, H2009, and H2087	5, 40 *µ*g/mL (A549) and 20 *µ*g/mL (H1299)	Bax:Bcl-2^↑^, caspase-9/-3^↑^, p-IKK-*β*^↓^, NF-*κ*B p65^┴^, TNF-*α*^┴^, ERK-1/2^┴^, JNK^↑^, PI3K/Akt^┴^, DDH^┴^, DRP-1^↓^, pro-caspase-3/-8^↑^, and Bax^↑^	[145, 153]
Breast	ZR-75-1, T47D, BT20, MCF-1, and MCF-7	—	DDH^┴^, DRP-1^↓^, pro-caspase-3/-8^↑^, and Bax^↑^	[153]
Gastric	AGS, SC-M1, NUGC-1, NUGC-3, and KOTA-III	—	DDH^┴^, DRP-1^↓^ pro-caspase-3/-8^↑^, and Bax^↑^	[153]

^↑^Upregulation; ^↓^downregulation; ^┴^inhibition.

## Abbreviations

Ache: Acetylcholinesterase
Bax: Bcl-2 associated protein
Bcl-2: B-cell lymphoma-2
COX-2: Cyclooxygenase-2
CXCL-1: Chemokine-1
CXCL-2: Chemokine-2
DAF-16: Abnormal dauer formation
DDH: Dihydrodiol dehydrogenase
DRP-1: Dynamin-related protein-1
E-cadherin: Epithelial cadherin
EMT: Epithelial to mesenchymal transition
Eotaxin-1: Eosinophil chemotactic protein
ERK: Extracellular signal-regulated kinase
GPX: Glutathione peroxide
GSH: Glutathione
HK2: Human kallikrein-related peptidase-2
IFN-*γ*: Interferon gamma
IgE: Immunoglobin E
IL-13: Interleukin-13
IL-1*β*: Interleukin-1 beta
IL-4: Interleukin-4
IL-6: Interleukin-6
IL-8: Interleukin-8
iNOS: Inducible nitric oxide synthase
I*κ*B*α*: Inhibitor of kappa B alpha
JNK: c-Jun N-terminal kinase
LC3A/B: Microtubule-associated protein 1 light chain 3A/B
MAPK: Mitogen-activated protein kinase
Mcl-1: Myeloid cell leukemia 1
MCP-1: Monocyte chemoattractant protein-1
MIP-1*α*: Macrophage inflammatory protein 2-alpha
miR-125: MicroRNA-125
MMP: Mitochondrial membrane potential
MMP-1: Matrix metalloproteinase-1
MMP-3: Matrix metalloproteinase-3
NCam: Neutral cell adhesion molecule
NO: Nitric oxide
PAR2: Protease-activated receptor 2
PGE2: Prostaglandin E2
PI3K: Phosphoinositide-3
PKC*β*2: Protein kinase C beta-2
PLC*γ*1: Phosphoinositide phospholipase C *γ*1
SAPK: Stress-activated protein kinase
SOCS-3: Suppressor of cytokine signaling 3
SOCS-5: Suppressor of cytokine signaling 5
SOD: Superoxide dismutase
SOD: Superoxide dismutase
SOX10: Sry-related HMg-Box gene 10
TGF-*β*1: Transforming growth factor beta 1
TLR-4: Toll-like receptor 4
TNF-*α*: Tumor necrosis factor alpha.
